# Contemporary Strategies in the Management of Civilian Abdominal Vascular Trauma

**DOI:** 10.3389/fsurg.2018.00007

**Published:** 2018-02-19

**Authors:** Georgios Karaolanis, Dimitrios Moris, C. Cameron McCoy, Diamantis I. Tsilimigras, Sotirios Georgopoulos, Chris Bakoyiannis

**Affiliations:** ^1^First Department of Surgery, Division of Vascular Surgery, National and Kapodistrian University of Athens, Athens, Greece; ^2^Department of Surgery, Duke University Hospital, Durham, NC, United States

**Keywords:** abdominal vascular trauma, penetrating injuries, blunt injuries, abdominal aorta injury, venous injuries, arterial injuries, open approach, endovascular approach

## Abstract

The evaluation and management of patients with abdominal vascular trauma or injury requires immediate and effective decision-making in these unfavorable circumstances. The majority of these patients arrive at trauma centers in profound shock, secondary to massive blood loss, which is often unrelenting. Moreover, ischemia, compartment syndrome, thrombosis, and embolization may also be life threatening and require immediate intervention. To minimize the risk of these potentially lethal complications, early understanding of the disease process and emergent therapeutic intervention are necessary. In the literature, the management of acute traumatic vascular injuries is restricted to traditional open surgical techniques. However, in penetrating injuries surgeons often face a potentially contaminated field, which renders the placement of prosthetic grafts inappropriate. Currently, however, there are sparse data on the management of vascular trauma with endovascular techniques. The role of endovascular technique in penetrating abdominal vascular trauma, which is almost always associated with severe active bleeding, is limited. It is worth mentioning that hybrid operating rooms with angiographic radiology capabilities offer more opportunities for the management of this kind of injuries by either temporary control of the devastating bleeding using endovascular balloon tamponade or with embolization and stenting. On the other hand, blunt abdominal injuries are less dangerous and they could be treated at most times by endovascular means. Since surgeons continue to encounter abdominal vascular trauma, open and endovascular techniques will evolve constantly giving us encouraging messages for the near future.

## Introduction

The evaluation and management of patients with abdominal vascular trauma or injury requires rapid and effective decision-making in these unfavorable circumstances. Penetrating abdominal trauma is by far the most common and accounts for about 90% of the cases (1, 2). The mortality rate varies widely and may reach 90% (3, 4). More than 70% of deaths can be expected to occur within the first day, whereas late-stage mortality may be attributed to secondary complications, such as sepsis and/or multiple organ failure, due to trauma (4). For this reason, emergent transfer to a trauma center, early assessment of the injury, and surgical intervention are critical for optimizing patient survival (1). Although, the management of acute traumatic vascular injuries was, until recently, restricted to traditional open surgical techniques (1, 2), the use of endovascular techniques provides a reliable alternative (5–8). In this review, we summarize all the available data on abdominal vascular trauma after the introduction of endovascular surgery in the treatment armamentarium, in order to provide surgeons and other physicians with a succinct and focused update.

## Surgical Anatomy

The major sites of hemorrhage in patients, victims from blunt or penetrating abdominal trauma, are the viscera, following the mesentery, and the major abdominal vessels (9). For a better estimation and treatment of the injuries, the abdomen was conventionally divided into three zones as follows (10):

- *Zone 1*: midline retroperitoneum (extending from the aortic hiatus to the sacral promotore). This zone is subdivided into the supramesocolic [suprarenal aorta, celiac axis (CA), superior mesenteric artery (SMA), renal arteries (RAs), the supramesocolic area of inferior vena cava (IVC), superior mesenteric vein (SMV)] area and the inframesocolic area that contains the infrarenal aorta and the IVC.
- *Zone 2*: upper lateral retroperitoneum (left and right, which contains the kidneys and their vessels)
- *Zone 3*: pelvic retroperitoneum (including the iliac vessels)

Recently, Feliciano et al. (11) reported a fourth zone in the perihepatic area that includes the hepatic artery, the portal vein (PV), the retrohepatic IVC, and the hepatic veins.

## Clinical Presentation

The clinical presentation is variable based on various parameters, such as the event, the involved vessel, the size of the injury, the presence of associated injuries, and the time elapsed since the injury. Abdominal vascular trauma can be presented in one of three ways: as free intraperitoneal hemorrhage, intraperitoneal/retroperitoneal hematoma, or thrombosis of the vessel (12). Under these circumstances, the hemodynamic status of the patients should be rapidly evaluated in order to divide them in two groups, those with ongoing hemorrhage and those without (hematoma/thrombosis). In cases of active hemorrhage, the patients arrive at the emergency department hypotensive and “non-responding” to the infusion of crystalloids and blood due to the presence of active bleeding directly into the peritoneal and/or retroperitoneal cavity. In this critical situation, the patients should undergo rapid assessment and transfer to an operating room for definitive repair of their vascular injuries. In cases of hematoma or thrombosis, their clinical status may be different. They may have only modest hypotension, and they are candidates for further imaging studies, as described below (12).

## Diagnostic Evaluation

In most critically ill patients with penetrating abdominal injuries, emergent laparotomy without additional investigations is needed. Rapidly obtained plain X-ray evaluation is of diagnostic value if available, because the location of the missile may be useful in designing the operation (9, 13). Multi-slice computerized tomography (CT) should be strongly considered as diagnostic tool to facilitate initial management decisions in more stable patients. Active bleeding after penetrating injuries is detected as a linear or irregular area of extravascular contrast-enhanced blood (14). Known drawbacks are the radiation exposure and the reactions to contrast material while the time required for transporting and performing CT scanning does not permit its use in unstable patients.

In patients with blunt trauma, radiographic diagnosis of bone injuries may increase the suspicion of vascular injuries. Under these circumstances, CT represents a useful tool for identifying large hematomas, false aneurysms, and/or vessel occlusion. In addition, angiography is an important tool in both diagnostic and therapeutic approaches for patients with blunt trauma. The main sites of application are the infrarenal aorta, RAs, and/or the iliac arteries. However, Maturen et al. (15) suggested that angiography has lower sensitivity and specificity compared to CT in active bleeding situations.

### Zone I Injuries

The IVC is the most commonly injured abdominal vessel and accounts for about 25% of abdominal vascular injuries (1). Penetrating injuries are by far the most common and account for 90% of the IVC injuries. Treatment of IVC injuries includes direct repair, patch repair, interposition of vein grafts, atrio caval shunting, and packing. Anterior perforations are repaired best using a continuous suture (venorrhaphy). In some patients with concomitant anterior and posterior perforation of the IVC, the posterior wound can be exposed and repaired from inside the vessel by extending the anterior wound (16). This approach is carried out when the patient is stable. When the patient is hemodynamically unstable with active bleeding or severe infrarenal injuries, or when repair produces stenosis, ligation is a first-option treatment and well tolerated (17). However, caution is required due to the likelihood of appearance of compartment syndrome in the lower limbs. Measurement of the pressures in the anterior compartment of the legs, bilateral fasciotomies if needed, maintaining the circulating volume stable in the postoperative period, and use of elastic compression wraps are some of the main strategies to assist this group of patients (17). Recently, in a review with 100 IVC injuries, ligation was performed in 25 cases with good results and without trace of lower extremity edema (17). However, there have been occasional reports of severe edema in postoperative period that has required later interposition grafting (18). On the other hand, ligation of the suprarenal IVC is not an acceptable option, because it results in renal failure. In these situations, common approaches are the application of a large venous patch taken from either the superior mesenteric or the ovarian vein and the application of a PTFE patch. However, this approach is rarely successful because of the patient’s poor hemodynamic status (18).

In the last few years, several reports (Table 1) on endovascular techniques based on the management of these complex injuries have been reported. Castelli et al. (19) reported their experience in a patient with blunt trauma. The injury of the IVC revealed by CT angiography was treated using a stent graft. Unfortunately, the patient died due to traumatic brain injury on post trauma day 2. Three other cases with a same similar management approach have recently been published (20–22). The mortality rate of patients, who arrive at the hospital with IVC injuries, ranges between 20 and 57% (23–30). About half of the patients with IVC injuries die before reaching the hospital and before any medical intervention.

**Table 1:** Abdominal veins involved in abdominal trauma: a summarizing table.

Reference	Year	Type of study	Number of patients (*N*)	Mechanism of injury	Injured vessel	Surgical approach	Endovascular approach	Mortality rate %
van Rooyen et al. (24)	2015	RS	27	PI	IVC	+		33
Sullivan et al. (17)	2010	RS	100	PI	IVC	+		59
Castelli et al. (19)	2005	CR	1	BI	IVC		+	100
Erzurum et al. (20)	2003	CR	1	BI	IVC		+	0
Watarida et al. (21)	2002	CR	1	BI	IVC		+	0
Tyburski et al. (117)	2001	RS	470	PI, BI	IVC, PV, HV, IV	+		45
Asensio et al. (1)	2000	RS	302	PI, BI	IVC, SMV, IMV, IV	+		IVC (75%)/SMV (19%)/IMV (25%)/RV (55.8%), IV (44.2%)
Ombrellaro et al. (28)	1997	RS	27	PI, BI	IVC	+		48
Fox et al. (62)	1996	CR	1	BI	IVC	+		0
Klein et al. (30)	1994	RS	38	PI, BI	IVC	+		21
Burch et al. (134)	1988	RS	577	PI	IVC, IV	+		37
Wiencek et al. (26)	1988	RS	67	PI	IVC	+		57
Ivatury et al. (103)	1987	RS	14	PI, BI	PV	+		50
Kudsk et al. (27)	1984	RS	70	PI, BI	IVC	+		88
Millikan et al. (25)	1983	RS	58	PI, BI	IVC, IV	+		38
Sirinek et al. (107)	1983	RS	5	PI, BI	IVC, PV, IV	+		32
Stone et al. (102)	1982	RS	41	PI, BI	PV	+		53
Kashuk et al. (91)	1982	RS	123	PI, BI	IVC	+		NR
Busuttil et al. (100)	1980	RS	21	PI, BI	PV	+		0
Petersen et al. (99)	1979	RS	28	PI, BI	PV	+		60
Pachter et al. (98)	1979	RS	11	PI, BI	PV	+		45.4
Graham et al. (29, 90)	1978	RS	301	PI, BI	IVC	+		100

*RS, retrospective study, CR, case report, PI, penetrating injury, BI, blunt injury, IVC, inferior vena cava, PV, portal vein, HV, hepatic vein, IV, iliac vein; NR, non reported*.

The abdominal aorta represents the second most common site of injuries (21%) reported after the IVC (25%) (1). For better estimation and approach, aorta injuries were classified based on CT findings and the presence of free rupture during laparotomy. Azizzadeh et al. (31) initially and Starnes et al. later (32) proposed a classification based on the alteration of the symmetric aortic shape observed in CT as follows:

- Intimal tear/minimal aortic injury: absence of aortic external contour abnormality and intimal defect and/or thrombus of <10 mm in length or width.
- Large intimal flap: absence of aortic external contour abnormality and intimal defect and/or thrombus of ≥10 mm in length or width
- Pseudoaneurysm: external contour abnormality and contained rupture
- Rupture: external contour abnormality with free contrast extravasation or hemoperitoneum found upon laparotomy

In a review of abdominal gunshot injuries, 2.7% were localized in the abdominal aorta (33). The infrarenal part was found injured in 50% of the patients, the supraceliac in 25%, and the remained space between celiac trunk and RAs was injured in 25% of patients (33, 34). Blunt abdominal aortic injury is rare and is related to biomechanical direct and indirect forces that take effect on the abdominal aorta, situated between the spinal column and the peritoneum and abdominal viscera. Intimal dissection is the result of these forces that can also lead to aortic transection. Moreover, thrombosis in the abdominal aorta due to the same mechanism or as a complication of aortic dissection has also been reported (34, 35).

The management of aortic injuries is complex and depends on various factors, such as the type, size, and location of the injury. Penetrating aortic injuries obviously require open repair due to the rapid extravasation of blood in the peritoneal and/or retroperitoneal cavity, which leads to life-threatening conditions. In case of blunt trauma, management may be less urgent. Blunt aortic injuries with small intimal tears could be managed conservatively, with closely follow-up (36–43). In cases of large intimal flaps or free rupture, open (1, 2, 10, 34, 36, 41, 43–63) or endovascular repair are required (5, 6, 10, 36, 38, 41, 60, 64–78) (Table 2).

**Table 2:** Studies referring to abdominal aorta and iliac artery trauma.

Reference	Year	Type of study	Number of patients (*N*)	Mechanism of injury	Injured vessel	Surgical approach	Endovascular approach	Conservative approach	Mortality rate
Garcia Reyes and Bellmunt Montoya (78)	2017	CR	1	BI	AA		+		0
Papazoglou et al. (64)	2015	CR	1	BI	AA		+		
de Mestral et al. (37)	2012	RS	42	BI	AA	+	+	+	6.9–7.1
Shalhub et al. (10)	2012	RS	28	BI	AA	+	+		32
Kawai et al. (65)	2010	CR	1	BI	AA		NBCA		0
Heck and Bittles (38)	2009	CR	1	BI	AA			+	0
Sakran and Mukherjee (66)	2009	CR	1	BI	AA		+		100
Huang et al. (39)	2009	CR	1	BI	AA		+	+	0
Jongkind et al. (44)	2009	CR	1	BI	AA, IA	+			0
Burjonrappa et al. (40)	2008	CR	1	BI	AA			+	0
Nucifora et al. (45)	2008	CR	1	BI	AA	+			100
Amini (46)	2008	CR	1	BI	AA	+			0
McCarthy et al. (47)	2007	CR	1	BI	AA	+			0
Gunn et al. (67)	2007	CR	1	BI	AA		+		0
Sugimoto et al. (48)	2007	CR	1	BI	AA	+			100
Rubin et al. (68)	2006	CR	1	BI	AA		+		0
Marti et al. (69)	2006	CR	1	BI	AA		+		0
Halkos et al. (70)	2006	CR	1	BI	AA		+		0
Choit et al. (41)	2006	CS	3	BI	AA, IA	+		+	0
Aidinian et al. (71)	2006	CR	1	BI	AA		+		0
Diaz et al. (49)	2006	CR	1	BI	AA	+			0
Lalancette et al. (50)	2006	CR	1	BI	AA	+			0
Vuorisalo et al. (72)	2005	CR	1	BI	AA		+		0
Teruya et al. (73)	2005	CR	1	BI	AA		+		0
Raghavendran et al. (51)	2004	CR	1	BI	AA	+			0
Muniz and Haynes (74)	2004	CR	1	BI	AA		+		0
Stahlfeld et al. (75)	2004	CR	1	BI	AA		+		0
Berthet et al. (42)	2003	RS	7	BI	AA	+	+	+	0
Meghoo et al. (52)	2003	CR	1	BI	AA	+			0
Rosengart et al. (53)	2002	CR	1	BI	AA	+			0
Inaba et al. (36)	2001	CS	4	BI	AA	+		+	0
Voellinger et al. (76)	2001	CR	1	BI	AA		+		0
Asensio et al. (1)	2000	RS	302	88% PI/12% BI	AA, IA	+			54
Kory (54)	2000	CR	1	BI	AA	+			0
Harkin et al. (55)	1999	CR	1	BI	AA	+			100
McEwan et al. (56)	1999	CR	1	BI	AA	+			0
Picard et al. (5)	1998	CS	3	BI	AA, IA		+		0
Qureshi et al. (58)	1997	CR	1	BI	AA	+			0
Siavelis and Mansour (59)	1997	CR	1	BI	AA, IA	+			0
Vernhet et al. (6)	1997	CS	3	BI	AA		+		0
Demetriades et al. (13)	1997	RS	224	PI	Abdominal Vessels	+		+	NR
Degiannis et al. (83)	1997	RS	57	PI	AA	+			85
Marty-Ane et al. (77)	1996	CR	1	BI	AA		+		0
Michaels et al. (60)	1996	RS	7	BI	AA	+	+		14.2
Tracy et al. (61)	1996	CR	1	BI	AA	+			0
Fox et al. (62)	1996	CR	1	BI	AA	+			0
Lopez-Viego et al. (34)	1992	RS	129	PI	AA	+			62
Frydenberg et al. (43)	1990	CS	4	BI	AA, IA	+		+	0
van Reedt Dortland and Clevers (63)	1988	CR	1	BI	AA	+			0

*RS, retrospective study, CR, case report, CS, case series, BI, blunt injury; PI, penetrating injury, AA, abdominal aorta, IA, iliac artery, NR, non reported, NBCA, n-butyl cyanoacrylate*.

Traditionally, aortic injuries are approached with the division of the midline of the retroperitoneum by the transverse mesocolon into the supramesocolic and inframesocolic regions. In the supramesocolic area, when hematoma is present, the surgeon has the time to mobilize all left-sided intra-abdominal viscera, including the colon, kidney, spleen, tail of the pancreas, and fundus of the stomach to the midline (left-sided medial visceral rotation) (79). This technique permits extensive exposure and visualization of the entire abdominal aorta from the aortic hiatus of the diaphragm to the aortic bifurcation. Drawbacks of this technique include the risk to damage the spleen and/or left kidney, accessories vessels originate from kidney, and the time to complete the maneuver (80). The bilateral subcostal abdominal approach with left medial rotation (“roof-top” approach) has been described as an alternative technique for treating various complex abdominal aortic pathologies. The main advantage is the avoidance of entering into the left thoracic cavity and that it may potentially be an attractive technique in cases of injuries in supramesocolic region (81). In the presence of active bleeding, immediate priority for the surgeon is the control of the bleeding by direct compression. Once this critical step is achieved, the next thought is to identify the bleeding vessel and to obtain proximal and distal control. However, due to the dense concentration of the major vessels (abdominal aorta, CA, SMA) in this area and the dense nature of the celiac plexus that surrounds the supraceliac aorta, in some cases, left thoracotomy traditionally seems the only safe way to obtain proximal aortic control (82). Injuries restricted within the inframesocolic area could be approached by retracting the transverse colon cephalad and mobilizing the small bowel to the right (79).

Supramesocolic injuries have a significantly worse outcome than inframesocolic injuries due to the viscera’s location, which makes aortic exposure challenging (34). The prognosis is better in case of blunt abdominal aortic trauma compared to penetrating trauma (37). Overall mortality after blunt and penetrating aortic injuries is estimated at 30 and 85%, respectively (37, 83, 84).

Endovascular management is used in selected cases, mainly in blunt aortic trauma. Any significant injury may be managed, if amenable, with stenting (64–78) or embolization (65). Unfortunately, existing data are limited to case reports (CRs) (Table 2) that describe injuries like limited infrarenal dissection and large intimal flaps (64–68, 70–78). Resuscitative Endovascular Balloon Occlusion of the Aorta (REBOA) offers a new tool for control of non-compressible abdominal vascular injuries. Recent advances in device technology have permitted for rapid deployment through smaller delivery (7 Fr) systems without dependence on fluoroscopy (85). Current guidelines through the American College of Surgeons find REBOA to represent a less invasive means of providing thoracic aortic occlusion compared to left thoracotomy with cross clamp to decrease blood loss from abdominal hemorrhage. Pelvic hemorrhage may also be selectively controlled through inflation of the device in the infrarenal aorta (86). While these two deployment strategies are promising, no data are currently available to demonstrate a mortality benefit compared to traditional techniques of initial vascular control. With this new technology comes consideration of new potential complications including femoral arterial injuries as well as extremity ischemia. Despite these points, REBOA offers a novel, non-invasive means of obtaining rapid vascular control in the exsanguinating abdominal trauma patient (85).

Injury of the CA is rare and may occur at the main trunk or any of its branches (1). Since Patman et al. (87) reported the first case, few studies (88–95) are available in the literature (Table 2). Asensio et al. (1) presented the largest series in the literature, in which 12 patients suffered from CA injuries after penetrating trauma. Eleven were treated with ligation and one with primary repair. The authors concluded that patients are not amenable to simple arteriorrhaphy should undergo ligation, which should not cause any short morbidity other than the risk of gallbladder necrosis (1). However, few data exist in the literature describing the consequences of this procedure, and surgeons should not worry about ligating the hepatic artery proper proximal to the origin of gastroduodenal artery, since the collateral flow through this vessel will maintain the viability of the liver. The reported mortality rate ranges from 38 to 75% (87, 88, 90–92). Therefore, the hemodynamic status of the patient, the complexity of the indicated operation (direct repair or conduit/graft needed for repair) and the associated injuries taking priority on operation plan should contribute to the final decision-making of repairing the celiac artery.

Injury to the PV trunk is relatively rare. Penetrating injuries are responsible for about 90% of the cases, as they are confirmed after laparotomy (96). The major percentage of patients, victims of PV penetrating injuries, present signs of hemorrhagic shock and require emergency laparotomy (97). On the other hand, blunt trauma often provokes thrombosis of the vessel and occasionally avulsion and bleeding (97).

Due to the location (retro/suprapancreatic) of the PV, the friability of its wall and the greater volume of blood through it, the management of this vessel injury is challenging. Exposure of retro-pancreatic PV and its major branches can be achieved by mobilization and medial rotation of the right colon and hepatic flexure of the colon, in association with extensive Kocher mobilization of the duodenum (26, 91). More often, stapled division of the neck of the pancreas is necessary for successful exposure (98–100). The same technique for dissection of the suprapancreatic PV has also been described (98, 99). There are several PV repair techniques, but lateral venorrhaphy is preferred (98, 99). Complex reconstruction using resection with end-to-end anastomosis, interposition grafting, transposition of splenic vein down to the SMV to replace the proximal PV, an end-to-side portocaval shunt, and a veno-venous shunt from the SMV to the distal PV or IVC have also been described (26, 91, 98–100). In addition, ligation of the vein is another surgical option compatible with survival, but in such cases bowel edema and wall necrosis are often complications. The experience after portal ligation is restrictive in the literature; however, there is no evidence of development portal hypertension in such cases (91, 98, 99). The mortality rate in PV injuries is high and ranges between 50 and 72% (101–103).

Several studies on SMA injuries have been conducted at experienced trauma centers (1, 84, 87, 91–93, 104–115) (Table 2). Since Fullen et al. (115) proposed an anatomic classification of injuries to the SMA, their management depends on the level of injury. Injuries in zone 1 (trunk proximal to the inferior pancreaticoduodenal artery) and zone 2 (between the inferior pancreaticoduodenal artery and the middle colic artery) can be achieved by left-sided medial visceral rotation and dissection anteriorly to the left kidney. Then the anterior aspect of the aorta including the SMA will be visible (79). However, these zones are characterized by high level of surgical difficulties, and under active bleeding in an unstable patient, the use of intraluminal shunt into the debrided ends of SMA should be considered. If the patient is stable and the proximal part of the SMA should be replaced, saphenous vein or prosthetic grafts have been described (84). On the other hand, injuries in zones III (distal to middle colic artery) and IV (the segmental intestinal branches) should be approached directly without any additional surgical manipulation.

Injuries of the inferior mesenteric artery (IMA) are rare. All the published studies (1, 87, 88, 93) reported results of IMA associated with other visceral vessels (Table 3). The main described mechanism was penetrating injury and accounted for 1% of all abdominal injuries (1). In critical situations, experts proposed ligation of the IMA with remarkable results for the bowel due to the rich collateral blood supply (1, 93).

**Table 3:** Summarizes the main studies with visceral arteries’ trauma.

Reference	Year	Type of study	Number of patients (*N*)	Injured vessel
Lopera et al. (131)	2011	RS	8	RA
Chabrot et al. (129)	2010	CS	3	RA
Bland et al. (95)	2006	CR	1	CA
Sangthong et al. (126)	2006	RS	517	RA
Memon and Cheung (130)	2005	CR	1	RA
Lee and White (8)	2002	CR	1	RA
Kavic et al. (94)	2001	RS	1	CA
Hagiwara et al. (123)	2001	RS	8	RA
Davis et al. (93)	2001	RS	18	CA, SMA, IMA
Villas et al. (125)	1999	CR	1	RA
Goodman et al. (128)	1998	CR	1	RA
Demetriades et al. (13)	1997	RS	224	Abdominal vessels
Whigham et al. (124)	1995	CR	1	RA
Jackson et al. (105)	1992	RS	2	SMA
Collins et al. (106)	1988	RS	6	SMA
Accola et al. (84)	1986	RS	22	SMA
Sclafani and Becker (122)	1985	RS	8	RA
Adkins et al. (92)	1985	RS	4	CA, SMA
Courcy et al. (104)	1984	RS	6	SMA
Sirinek et al. (107)	1983	RS	20	SMA
Kashuk et al. (91)	1982	RS	12	CA, SMA
Lucas et al. (108)	1981	RS	15	SMA
Ekbom et al. (109)	1981	RS	5	SMA
Phillips et al. (110)	1979	RS	1	SMA
Graham et al. (29, 90)	1978	RS	64	CA, SMA, IMA
Mattox et al. (96, 112)	1975	RS	8	CA, SMA
Kelly and Eiseman (113)	1975	RS	4	SMA
Ledgerwood and Lucas (114)	1972	RS	1	SMA
Fullen et al. (115)	1972	RS	8	SMA
Perry et al. (89)	1971	RS	2	CA
Perdue and Smith (88)	1968	RS	12	CA, SMA, IMA
Patman et al. (87)	1964	RS	6	CA, SMA, IMA

*RS, retrospective study, CR, case report, CA, celiac artery, SMA, superior mesenteric artery, IMA, inferior mesenteric artery, RA, renal artery; IA, iliac artery; CS, case series*.

### Zone 2 Injuries

The management of renovascular injuries depends on the mechanism of injury, the time of diagnosis, the ischemia time, the general condition of the patient, and the presence of a contralateral normal kidney. Injuries to the renal vasculature (zone 2) are difficult to manage due to the small size of the vessel and its location deep in the retroperitoneum. Occasionally, small injuries after penetrating trauma can be repaired by lateral arteriorrhaphy or resection and end-to-end anastomosis (11, 93). Interposition grafting with saphenous vein, PTFE graft, and rarely harvesting of the splenic and hepatic arteries in order to replace the left/right RAs, respectively, has also been presented (116). However, the latter approach is not advisable under hemodynamic instability but when the patient is stable. In cases with penetrating wounds associated with hemodynamic instability and with significant renovascular injuries or long period of ischemia, nephrectomy may be the better choice, as long as a normal contralateral kidney was confirmed (11). The survival rate after penetrating injuries ranges between 65 and 87% (117) with renal salvage in only 30–40% of the cases (118, 119).

The management of blunt injuries to the RAs is complicated by the often-delayed diagnosis and prolonged ischemia of the kidney. The patients often complain about abdominal and flank pain, whereas associated signs like gross or microscopic hematuria are also reported. The management of isolated main RA occlusion remains controversial. Treatment options include immediate nephrectomy, non-operative management, or revascularization by surgical or endovascular techniques (11). In stable patients with short warm ischemia time (<5 h), revascularization should be performed (11, 16, 93). In cases of delayed diagnosis (>5 h), most surgeons avoid revascularization, unless the injury involves both kidneys or solitary kidney, due to the disappointing results after revascularization (120, 121). Other patients, assuming they have a normally functioning contralateral kidney, should be either monitored or considered for endovascular procedures. At least, patients with bilateral RA injuries or those with solitary kidney should be strongly considered for revascularization (11).

The advancement of endovascular techniques has opened new horizons in the management of renovascular injuries. However, experience with this technique is still limited (8, 122–127). First, Sclafani and Becker (122) reported eight patients with penetrating (*n* = 6) and blunt trauma (*n* = 2), who were treated with angiographic embolization. Seven of these eight patients underwent successful procedures with preservation of the renal function, and only one nephrectomy due to hematuria was performed. Later, another study (123) reported the same results after angiographic embolization, obviating the need for open surgery. All patients presented normal functioning status at discharge. In more recent years, few CRs (Table 3) document the successful use of various stents to obliterate intimal flaps after RA injuries without short-term complications (8, 124–131). Thus, endovascular treatment may play an important role in selected cases of blunt renovascular trauma, when patients are stable with injuries like intimal tears, acute occlusions, false aneurysms, and arteriovenous fistulae.

Injury of the renal vein is the result of penetrating and blunt trauma mechanisms. Although, blunt avulsion injuries result in exsanguination, under penetrating trauma, the patient may be stabilized with retroperitoneal tamponade (1). Lateral venorrhaphy remains the first treatment choice; however, it is not possible in case of extensive injuries. In these cases, ligation of the renal vein has been proposed unless the collateral vein circulation is inappropriate, to maintain stable and vital the kidneys. Ligation of the left renal vein is tolerated well due to satisfactory venous drainage through the left gonadal vein, left adrenal vein, and lumbar veins. This choice is not feasible in cases of the right renal vein, when the collateral vein flow is absent, and nephrectomy is the only choice (93). The survival rate ranged from 44 to 70% with a mean of 60% (1, 93).

### Zone 3 Injuries

Injuries of the iliac arteries due to penetrating or blunt trauma are described in the literature (Table 2). Penetrating trauma is by far the most common mechanism of injury, whereas blunt trauma remains uncommon; the only available data on blunt trauma come from CRs. Velmahos et al. (132) published their experience in 30 patients suffering from blunt injury to the iliac arteries. Seventeen patients (56.6%) underwent embolization of the bleeding internal iliac arteries as primary treatment while the rest of the patients had undergone to laparotomies before the embolization. The success rate was 97% (29–30) in controlling pelvic hemorrhage. Later, Cestero et al. (133) reported that iliac artery injury occurred after penetrating trauma in 10% of the cases, whereas 26% of the patients had combined arterial and venous injuries. The common involved site is the common iliac artery (CIA) and the branches of the internal iliac artery for penetrating and blunt trauma, respectively. Often the patient shows severe hypotension and abdominal distension in case of penetrating trauma and with signs of absent or diminished femoral pulses (1, 41, 44). Thrombosis is observed in later stages of blunt injuries through diagnostic imaging such as Duplex ultrasound and/or CT (41).

Surgical exploration should be carried out in cases of active bleeding after penetrating trauma and if blunt trauma persists with associated intraperitoneal leak, hematoma that creates absent or diminished femoral pulses or it has continuing expansion (11, 44). Arterial injuries could be repaired with vascular sutures (arteriorrhaphy) or interposition of venous/PTFE grafts. Special consideration should be taken to avoid graft infection, for which some authors proposed an extra-anatomic bypass as the method of treatment (134). On the other side, supporters of the endovascular technique proposed this approach in order to avoid complications due to the contamination of the graft.

Thus, endovascular treatment is a trustworthy alternative in cases of further graft infection and in cases with false aneurysm, arteriovenous fistulae, major intimal tears, and vessel’s thrombosis (135). Moreover, it was also proposed by some experts as the first-line therapeutic option due to the low complication rate in cases of chronic traumatic injuries of the CIA or external iliac artery (7, 136).

Iliac venous injuries are technically challenging due to the difficult exposure caused by anatomic placement behind the arteries. Injuries on the common and/or external iliac veins could be managed with lateral repair with polypropylene sutures (4-0, 5-0) or with ligation (1). In case of significant narrowing after lateral repair, treatment with anticoagulants is appropriate to reduce the risk of thrombosis and/or pulmonary embolism. Ligation is usually well tolerated although many patients develop transient leg edema. Complex reconstruction with spiral grafts or prosthetic materials is not recommended.

The traditional pre-peritoneal pelvic packing represents a trustworthy alternative to address venous hemorrhage in complex pelvic fractures (137). The “trigger” to perform this technique is the persistent hemodynamic instability of the patient despite two units of red blood cells transfusion during the initial resuscitation (137). Other proposed indications were unstable patients with pelvic hematoma diagnosed on focused assessment with sonography in trauma exam and in cases of unavailable angiographic embolization in some centers (138). The survival rate of patients with iliac venous injuries ranges between 65 and 95% (1, 25, 93, 107, 117, 134).

## Treatment Algorithm

**Figure 1:**
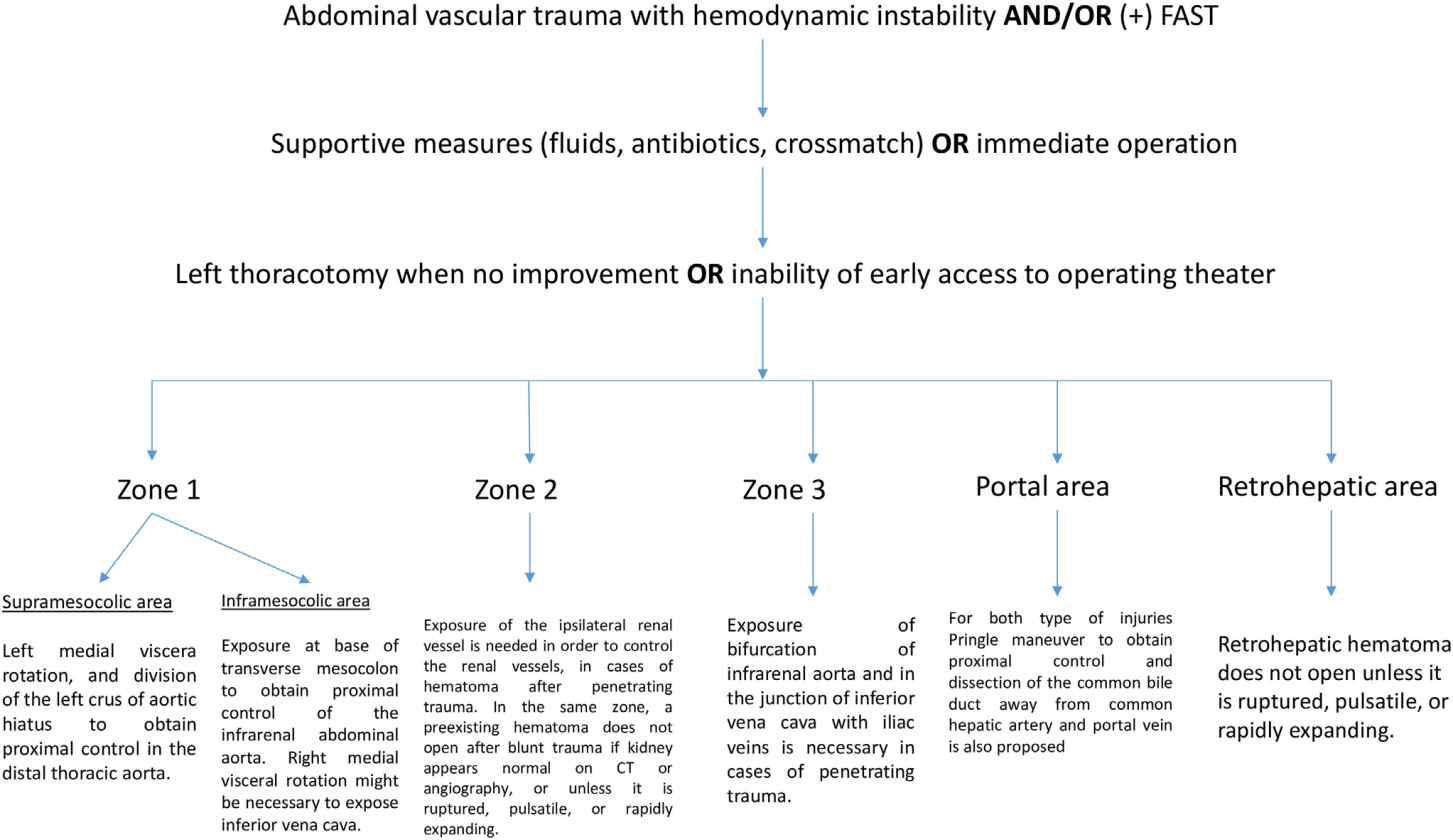
Algorithm of approaching abdominal vascular trauma.

Abdominal vascular injuries represent a devastating situation, requiring immediate and effective decision-making. Penetrating injuries are most common and present either active bleeding or a contained retroperitoneal, mesenteric, or portal hematoma. Recently, experts proposed a treatment algorithm based on the management of abdominal vascular trauma for better understanding and surgical approach (11). In brief, for penetrating injuries in supramesocolic area (zone 1), left medial viscera rotation and division of the left crus of aortic hiatus to obtain proximal control of the distal thoracic aorta have been proposed. Another means of proximal aortic control may be obtained adjacent to the esophagus and stomach through the lesser omentum. Hematoma located in inframesocolic area requires exposure at base of the transverse mesocolon to obtain proximal control of the infrarenal abdominal aorta by means of evisceration of the small bowel to the right and opening the retroperitoneum at base of transverse mesocolon. Unless aortic injury has been detected, right medial visceral rotation is necessary to expose IVC. For penetrating zone 2 injuries, exposure of the ipsilateral renal vessel is needed in order to control the renal vessels. In the same zone, a preexisting hematoma does not require exploration after blunt trauma if the kidney appears normal on CT or angiography. Active bleeding confined to zone 2 may be approached with division of lateral peritoneum and Gerota’s fascia whereas some situations dictate a medial to lateral approach for early control of the hilum. Figure 1 illustrates the algorithm described above. Exposure of the bifurcation of infrarenal aorta and in the junction of IVC with iliac veins is necessary in cases of penetrating trauma confined in zone 3.

## Conclusion

Abdominal vascular injuries are commonly seen in daily clinical practice. Active bleeding after penetrating injuries is the most dreadful scenario that surgeons have to face. Hematoma in most times spares time to the surgeon for better decision-making and surgical approach. Vascular repair are generally carried out with arteriorrhaphy and/or venorrhaphy or even with insertion of substitute vascular conduits. Endovascular technique gains more and more field in the management of blunt trauma and in most cases with delayed vascular complications, such as aneurysms, arteriovenous fistulae, and arterial occlusion. The role of endovascular technique in penetrating abdominal vascular trauma, which is almost always associated with severe active bleeding, is limited. It is worth mentioning that hybrid operating rooms with angiographic radiology capabilities offer more opportunities for the management of this kind of injuries by either temporary control of the devastating bleeding using endovascular balloon tamponade or with embolization and stenting. Abdominal vascular trauma continues to represent a difficult problem and, open and endovascular technique continue to evolve to address this complex disease process.

## Author Contributions

CB: organized the study and reviewed the manuscript. GK: gathered the data, organized the study, and drafted the manuscript. CM and DM: gathered the data. DT and SG: reviewed the manuscript.

## Conflict of Interest Statement

The authors declare that the research was conducted in the absence of any commercial or financial relationships that could be construed as a potential conflict of interest. The reviewer AML declared a shared affiliation, with no collaboration, with the authors to the handling Editor.
